# A Case of Multiple Sclerosis Uncovered Following Moderna SARS-CoV-2 Vaccination

**DOI:** 10.7759/cureus.32799

**Published:** 2022-12-21

**Authors:** Ange Ahoussougbemey Mele, Henry Ogbuagu, Sahil Parag, Bradley Pierce

**Affiliations:** 1 Internal Medicine, Northeast Georgia Medical Center Gainesville, Gainesville, USA

**Keywords:** sars-cov-2, covid-19 prevention, literature review of disease, multiple sclerosis flare-ups, multiple sclerosis, covid-19 vaccine

## Abstract

Multiple sclerosis is a demyelinating disorder of the central nervous system characterized by lesions disseminated in time and space. The diagnostic criteria for laboratory-supported definite multiple sclerosis involve two episodes of symptoms, evidence of at least one white matter lesion on MRI, and abnormal oligoclonal bands in cerebrospinal fluid. Patients usually present in their early 20s and on average have up to one flare-up per year. While vaccines play an important role in the prevention of many diseases, they have often been purported as a potential trigger of multiple sclerosis and multiple sclerosis relapses. The medical literature provides reliable information concerning the risk of developing multiple sclerosis and multiple sclerosis relapses following the administration of most vaccines, but not much is known about the novel Moderna severe acute respiratory syndrome coronavirus 2 (SARS‑CoV‑2) vaccine.

We report the case of a 24-year-old male who presented with right-sided facial weakness, dizziness, and dysarthria two days after receiving his first dose of the Moderna coronavirus disease 2019 (COVID-19) vaccine. Imaging studies noted both acute and chronic central nervous system lesions. He met the diagnostic criteria for laboratory-supported definite multiple sclerosis. His acute flare was treated with intravenous corticosteroids and the patient was subsequently started on ocrelizumab.

This case serves as an important example of the novel Moderna SARS-CoV-2 vaccine as a potential trigger of multiple sclerosis relapse. In addition, we review the literature for similar occurrences with the other COVID-19 vaccines and provide reliable guidance for COVID-19 vaccination for patients with multiple sclerosis.

## Introduction

With the advent of the coronavirus disease 2019 (COVID-19) pandemic, different vaccines have been developed to protect the population and curtail the increasing number of deaths from the virus. The Centers for Disease Control and Prevention has reported that patients with neurological disorders were at risk of severe illness from COVID-19. This statement was based on the evidence collected from reviews, cross-sectional studies, and cohort studies [1]. As part of this group, patients with multiple sclerosis were therefore given priority to receive the vaccine first. The vaccine is now available to everyone. Here, we report a case of multiple sclerosis relapse following the Moderna COVID-19 vaccine. We review the literature for similar occurrences and discuss the American Academy of Neurology (AAN) guidelines for COVID-19 vaccination of multiple sclerosis patients on different disease-modifying therapies.

## Case presentation

We report the case of a 24-year-old male with a medical history of obesity who presented to the Emergency Department for evaluation of right-sided facial weakness, dysarthria, and dizziness two days after receiving his first dose of the Moderna COVID-19 vaccine. He denied any previous similar presentation, headaches, chest pain, shortness of breath, and abdominal tenderness. Motor strength was 5/5 bilaterally in both upper and lower extremities, tandem gait was intact, and there were no sensory neurological deficit, dysdiadochokinesia, or abnormal Romberg sign. Cranial nerves were intact on examination. There was no evidence of facial droop, eyelid lag, and extraocular movement abnormalities. There was no evidence of bowel or bladder symptoms.

**Figure 1:**
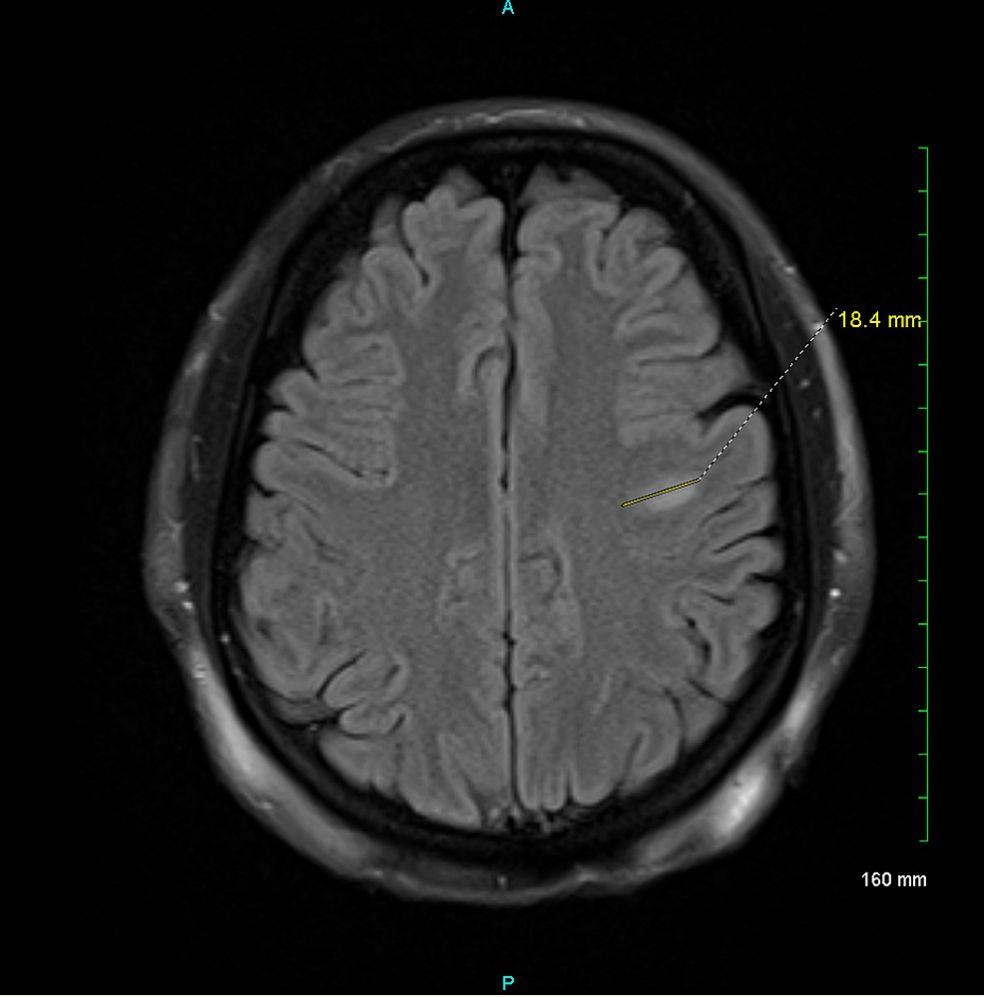
An ovoid 18 mm subcortical left frontal white matter hyperintense lesion with restricted diffusion and smudge-like contrast enhancement.

**Figure 2:**
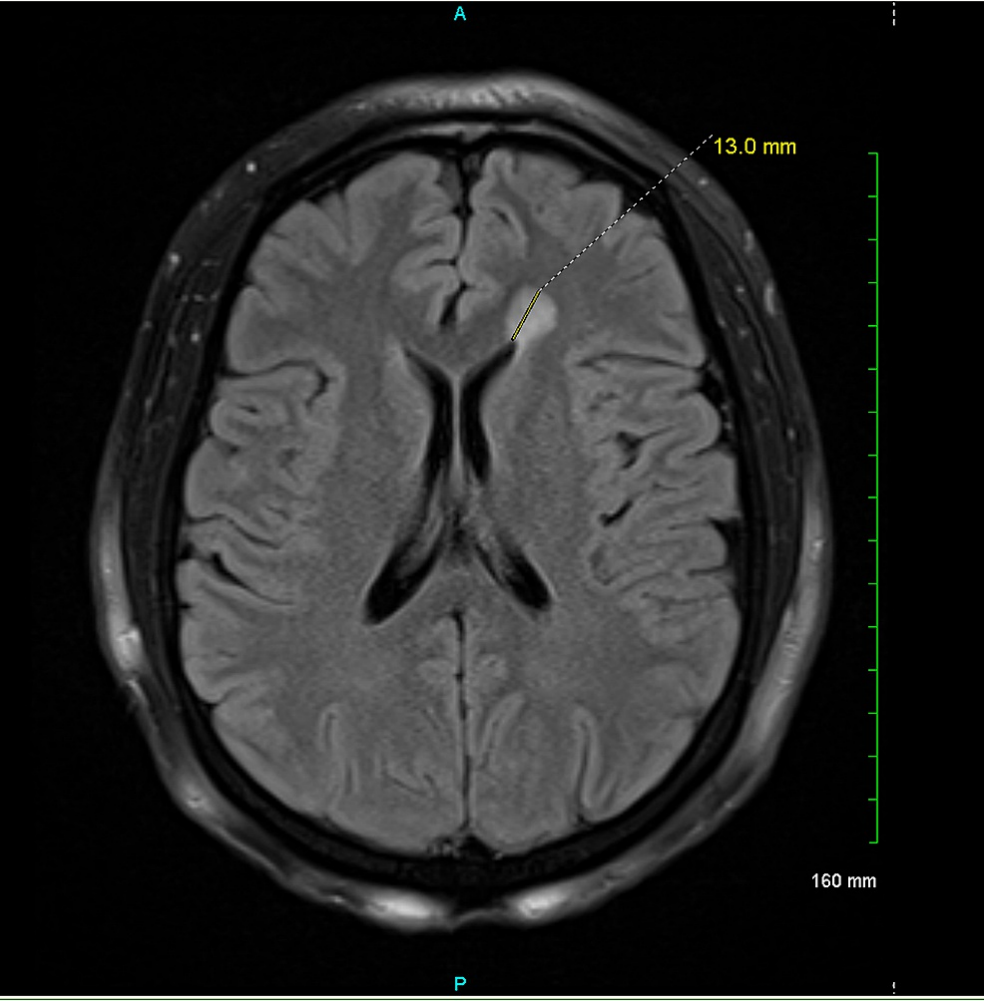
A round 13 mm T2 FLAIR signal hyperintense lesion in the left frontal white matter abutting the frontal horn of the left lateral ventricle. This lesion does not have restricted diffusion and lacks contrast enhancement. FLAIR = fluid-attenuated inversion recovery

His chest X-ray was negative for any acute cardiopulmonary disease, and his electrocardiogram was consistent with sinus rhythm with a rate of 89 beats per minute. A transthoracic echocardiogram showed normal left ventricular systolic and diastolic functions with an ejection fraction of 55-60%. A bubble study was positive for a trivial intracardiac shunt. CT angiography of the head and neck was negative for acute intracranial processes and did not show any focal narrowing of the cervical or intracranial arteries. MRI of the brain was notable for an active demyelinating lesion in the left frontal subcortical white matter (Figure 1). Additional regions of white matter signal abnormality, most notably involving the left frontal horn, were expected to be older regions of demyelination (Figure 2). There was no evidence of involvement below the tentorium cerebelli, and the optic nerves appeared normal.

At this point, his acute flare was treated with intravenous corticosteroids. The patient subsequently underwent a lumbar puncture. Meningitis and encephalitis polymerase chain reaction panel was unremarkable. His multiple sclerosis panel was positive for seven oligoclonal bands. His immunoglobulin G level in the cerebrospinal fluid (CSF) was within normal limits. The results of his CSF cell count with differential were all within normal limits. Lupus anticoagulant profile, antinuclear antibodies, and aquaporin-4 antibodies were negative. Myelin oligodendrocyte glycoprotein was not checked. The patient met the diagnostic criteria for definite multiple sclerosis and was diagnosed with the condition. He received the second dose of the Moderna vaccine one month and four days later without further side effects. He was subsequently started on ocrelizumab.

## Discussion

According to Olek, the 2017 McDonald criteria is the best method to establish the diagnosis of multiple sclerosis. In fact, these criteria require two relapses and two objective signs over time or one relapse plus two clinical signs and specific MRI findings to establish the diagnosis of multiple sclerosis. The clinician must also ensure there is no other explanation for the clinical and imaging findings [2].

The mechanism of action of the Pfizer-BioNTech and Moderna vaccines has been explained by Mascellino et al. According to this study, the mechanism of the mRNA vaccines is to introduce the code to make spike proteins from severe acute respiratory syndrome coronavirus 2 (SARS‑CoV‑2) which triggers a cellular immune response [3]. Before receiving the vaccine, our patient did not know he had multiple sclerosis. His MRI showed older regions of demyelination in the left frontal horn as well as a new active demyelinating lesion in the left frontal subcortical white matter. He developed a multiple sclerosis relapse after receiving the first dose of the mRNA vaccine. His acute flare was treated with intravenous corticosteroids, and he recovered promptly.

Maniscalco et al. have also reported a case of multiple sclerosis relapse in a 26-year-old female patient 48 hours after receiving the Pfizer-BioNTech vaccine. The patient presented with paresthesia in her left arm and limbs along with walking difficulties. The patient’s MRI showed three new voluminous enhancing lesions. She recovered after five days of methylprednisolone therapy. The authors call for further work to redefine the risk/benefit ratio of COVID-19 vaccination in patients with multiple sclerosis [4]. However, a study that implied a low risk/benefit ratio was conducted by Achiron et al. It was an observational study in one clinical center in Israel where 555 multiple sclerosis patients received the Pfizer-BioNTech vaccine. The safety profile was noted to be the same as in premarketing clinical trials where patients mostly experienced fatigue, headache, and injection site reactions. Acute relapses were detected in 2.1% of patients following the first vaccine dose and in 1.6% of patients after the second dose [5]. The authors noted increased risks of adverse effects in younger patients aged 18 to 55. This low rate of relapse might be explained by the short follow-up period of 20 and 38 days following the first and second vaccine administration, respectively.

To further corroborate the previous findings, Nistri et al. reported a series of 16 cases of multiple sclerosis relapses occurring between three days and three weeks following administration of the Pfizer vaccine in 10 patients, Moderna vaccine in two patients, and AstraZeneca in four patients between March and June 2021 [6]. Three of these 16 patients were newly diagnosed with multiple sclerosis after COVID-19 vaccination, 13 were known multiple sclerosis patients, and nine were being treated with disease-modifying therapies. All patients had evidence of newly active lesions on MRI. The authors stated that the causative or incidental nature of this relationship remains to be established.

Vaccines with other mechanisms of action have also been implicated in multiple sclerosis relapse, such as the Sputnik vaccine in Russia. Its mechanism of action involves two adenovirus viral vectors (recombinant-adenovirus 5 and 26). According to Etemadifar et al., a 34-year-old rituximab-treated multiple sclerosis patient, who was diagnosed with relapsing-remitting multiple sclerosis 13 years ago, developed hemiplegia and ataxia following administration of the Sputnik vaccine [7]. This occurred three months after his last rituximab infusion and three days following his first dose of the Sputnik vaccine. Moreover, the study further raised concerns about the safety and efficacy of the vaccine in multiple sclerosis patients treated with anti-CD20 monoclonal antibodies. The patient’s anti-SARS-CoV-2 antibodies were below the lower detectable limit. The patient, however, received the second dose of the Sputnik vaccine without additional side effects. The authors postulated that the efficacy of COVID-19 vaccination is limited in patients who are being treated with anti-CD20 monoclonal antibodies and recommended planning a delay in such treatments to enable patients to receive the vaccine and develop anti-SARS-CoV-2 immunity.

According to Bagnato et al., multiple sclerosis patients and those on immunosuppressive medications were excluded from clinical trials led by Pfizer-BioNTech. The Moderna study did not mention multiple sclerosis as a comorbidity and did not make it a part of the exclusion criteria either. The Johnson & Johnson phase 3 clinical trial is not fully public yet [8]. As such, the use of these vaccines in multiple sclerosis patients on disease-modifying therapies is mainly based on previous studies of other vaccines. Based on these studies, immunization, in general, is considered safe in people with multiple sclerosis. Moreover, the studies recommend receiving the vaccine if multiple sclerosis patients do not have any other known contraindications to doing so. The Multiple Sclerosis Centers of Excellence further postulated that COVID-19 vaccines were important and safe for veterans with multiple sclerosis [9].

Concerning MS patients who are on immunosuppressive medications, a discussion with their primary neurologist is recommended to understand the importance of vaccination, the minimal risks associated with it, and to decide if any treatment modification is necessary. Furthermore, Righi et al. stated that mRNA-based or inactivated vaccines are considered safe in multiple sclerosis patients undergoing immunomodulatory or immunosuppressive treatments [10]. Finally, according to guidance from the National Multiple Sclerosis Society (NMSS), discontinuing disease-modifying agents is associated with significant risks of relapse and worsening clinical course. As such, the NMSS recommends continuing these medications unless otherwise advised by their primary neurologist [11].

The AAN also released guidance on the vaccination of multiple sclerosis patients on different types of disease-modifying therapies [12]. The first recommendation is for patients on beta-interferons, glatiramer acetate, teriflunomide, dimethyl or monomethyl fumarate, or natalizumab. The AAN advised against discontinuing these disease-modifying agents and did not recommend a delay or adjustment in dosing or timing of administration of these medications. The second recommendation is for multiple sclerosis patients on anti-CD20 monoclonal infusions. Patients on these medications are prone to attenuation of the humoral response. Therefore, it is advised to be vaccinated >12 weeks after the last infusion and to resume infusion four weeks after the last dose of the vaccine to maximize the efficacy of the vaccine. The third recommendation concerns patients on alemtuzumab. Given its effects on CD52+ cells, it is advised to be vaccinated >24 weeks after the last infusion and to resume infusion four weeks after the last dose of the vaccine. Multiple sclerosis patients starting alemtuzumab are advised to be fully vaccinated first and start the medication four weeks or more after completing the vaccine. The fourth recommendation concerns multiple sclerosis patients on sphingosine 1 phosphate receptor modulators, oral cladribine, and ofatumumab. Multiple sclerosis patients starting these medications are also advised to be fully vaccinated and then start these disease-modifying agents two to four weeks after completing the vaccine. Patients are not advised to change the schedule of administration. When possible, however, patients should restart taking their doses of cladribine or ofatumumab two to four weeks after the last dose of the vaccine. These recommendations should be followed only when there is enough disease stability to allow delays in treatment. Complete blood count with differential should be collected to obtain an estimate of white blood cell and lymphocyte count in patients with a markedly suppressed immune system.

## Conclusions

Overall, it can be concluded that many cases of multiple sclerosis relapses have been reported following the administration of COVID-19 vaccines with different mechanisms of action. The NMSS and the Multiple Sclerosis Centers of Excellence advocate for the safety of the vaccine in multiple sclerosis patients. Moreover, the AAN has released guidelines for the vaccination of multiple sclerosis patients on different disease-modifying therapies. Further research needs to be done to better comprehend the mechanism of these relapses following the administration of the different COVID-19 vaccines. Meanwhile, following these guidelines, as well as continued patient education and clinical monitoring, will contribute to mitigating these incidental side effects.
